# The winter urban heat island: Impacts on cold-related mortality in a highly urbanized European region for present and future climate

**DOI:** 10.1016/j.envint.2021.106530

**Published:** 2021-09

**Authors:** Helen. L. Macintyre, Clare Heaviside, Xiaoming Cai, Revati Phalkey

**Affiliations:** a Climate Change and Health Group, Centre for Radiation Chemical and Environmental Hazards, Public Health England, Chilton, Oxon OX11 0RQ, UK; b School of Geography, Earth and Environmental Sciences, University of Birmingham, Edgbaston, Birmingham B15 2TT, UK; c Institute for Environmental Design and Engineering, University College London, Central House, 14 Woburn Place, London WC1H 0NN, UK; d Division of Epidemiology and Public Health, School of Medicine, University of Nottingham City Hospital, Hucknall Road, NG51PB Nottingham, UK; e Heidelberg Institute for Global Health, University of Heidelberg, Im Neuenheimer Feld 130.3, 69120 Heidelberg, Germany

**Keywords:** Urban health, Temperature exposure, Climate change, UHI, WRF

## Abstract

Exposure to heat has a range of potential negative impacts on human health; hot weather may exacerbate cardiovascular and respiratory illness or lead to heat stroke and death. Urban populations are at increased risk due to the Urban Heat Island (UHI) effect (higher urban temperatures compared with rural ones). This has led to extensive investigation of the summertime UHI and its effects, whereas far less research focuses on the wintertime UHI. Exposure to low temperature also leads to a range of illnesses, and in fact, in the UK, annual cold-related mortality outweighs heat-related mortality. It is not clearly understood to what extent the wintertime UHI may protect against cold related mortality.

In this study we quantify the UHI intensity in wintertime for a heavily urbanized UK region (West Midlands, including Birmingham) using a regional weather model, and for the first time, use a health impact assessment (HIA) to estimate the associated impact on cold-related mortality.

We show that the population-weighted mean winter UHI intensity was +2.3 °C in Birmingham city center, and comparable with that of summer. Our results suggest a potential protective effect of the wintertime UHI, equivalent to 266 cold-related deaths avoided (~15% of total cold-related mortality over ~11 weeks). When including the impacts of climate change, our results suggest that the number of heat-related deaths associated with the summer UHI will increase from 96 (in 2006) to 221 in the 2080s, based on the RCP8.5 emissions pathway. The protective effect of the wintertime UHI is projected to increase only slightly from 266 cold-related deaths avoided in 2009 to 280 avoided in the 2080s. The different effects of the UHI in winter and summer should be considered when assessing interventions in the built environment for reducing summer urban heat, and our results suggest that the future burden of temperature-related mortality associated with the UHI is likely to increase in summer relative to winter.

## Highlights

• Mean city centre winter UHI intensity was +2.3 °C reaching maximum of +9.9 °C.
• Winter UHI reduces cold-related mortality by 15% (266 deaths avoided).
• This compares with increased mortality of 36% (96 deaths) in summer.
• Winter UHI continues to have a protective effect in future climate.

## 1 Introduction

Hot or cold weather can have negative impacts on human health, and can exacerbate conditions such as cardiovascular and respiratory diseases, leading to increased risk of hospitalization and death (Basu and ambient temperature and mortality: a review of epidemiologic studies from, , 2001, Gómez-Acebo et al., 2013, Guo et al., 2014). Temperature may be influenced locally by the Urban Heat Island effect (UHI), where replacement of natural land surfaces with urban materials such as buildings, roads, and other paved areas leads to locally increased temperatures in towns and cities, when compared with rural areas (Oke, 1982). The UHI can contribute to thermal discomfort, and a range of morbidity and mortality outcomes (Heaviside et al., 2017). Most research on the health impacts of the UHI focus on summer; for example, estimates are that 40% of heat-related mortality in the West Midlands, UK, could be attributed to the UHI intensity during the summer of 2006, and up to 50% during the heatwave of 2003 (Heaviside et al., 2016, Macintyre and Heaviside, 2019).

Despite a growing risk to health from heat-related mortality (due to climate change), in the UK cold effects on health generally outweigh those from heat, with estimates suggesting ~41,000 cold-related deaths annually, compared with ~2,000 heat-related deaths for the 2000s (Hajat et al., 2014). Projections suggest that UK mean air temperatures could increase by between 0.7 °C and 4.2 °C in winter, and 0.9 °C to 5.4 °C in summer by 2070 (RCP8.5, unmitigated emissions pathway) (Lowe et al., 2018). Previous work has shown that heat-related mortality could increase from ~2000 per year in the 2000s to ~7000 deaths per year by 2050 in the UK due to the effects of climate change, while there is relatively little reduction in cold-related mortality, partly due to population changes (Hajat et al., 2014, HPA, 2012). The combined risks of increases in temperature due to climate change, the UHI effect, and the increased fraction of the population living in urban areas (82% in the UK, (ONS, 2011)) means that urban populations are at particular risk from heat. As such, it is understandable that much of the research on temperature-related health effects and UHIs is dominated by the summer season, with the winter UHI less well studied. It is reasonable to expect that any increased temperature effect due to the UHI in winter could potentially have a protective impact on health, i.e. in reducing cold-related mortality, although this has not been explicitly tested to date. Even with climate change, the considerable mortality burden related to cold is expected to continue in future; this motivates a study on the role of the UHI in modifying cold-related mortality in the UK.

Studies suggest the average intensity of the UHI varies with season, with summer UHIs being slightly larger (1 °C to 4 °C) than winter UHIs (1 °C to 3 °C) (Gedzelman et al., 2003, Imhoff et al., 2010, Kłysik and Fortuniak, 1999, Runnalls and Oke, 2000, Yang and Bou-Zeid, 2018). While in summer there is a large diurnal cycle in the UHI, it appears to vary less in winter. The generally greater UHI intensity in summer is driven by the longer days and higher incident sun angles at this time of year, which lead to more solar radiation being absorbed and subsequently released from urban structures; the effect is particularly strong at night-time in summer. Seasonal variation in UHI may also be influenced by the specific locations of the city (for example cities under coastal influence such as Vancouver), and autumnal fog may drive large urban–rural temperature differences around sunrise (Runnalls and Oke, 2000). In the UK, London’s UHI in winter has been estimated (using an array of temperature sensors) to be 0.6 °C to 1.2 °C during the day, and 0.6 °C to 1.7 °C at night, depending on the amount of wind and cloud (Giridharan and Kolokotroni, 2009); this is similar but slightly smaller than the summer UHI estimated by the LUCID project (Mavrogianni et al., 2011). Another study estimated the UHI at a single point in London to be 4 °C overnight in winter, with little difference during the day, based on simulation of a single 24-hour period (10/11 December 2009) (Bohnenstengel et al., 2014). Using monthly minimum values of air temperatures estimated using a combination of observations and regression modelling from 1961 to 2006 at 5 km resolution, the winter UHI in UK cities was estimated at 1 °C to 2 °C, while in summer it was 2 °C to 3 °C (Kershaw et al., 2010). However, compared to the UHI in summer, there is a clear gap in understanding related to the wintertime UHI and how it may influence cold-related mortality, and how these effects and impacts may change with climate change.

The relationship between high or low temperatures and health effects typically follows an approximate ‘U’ or ‘J’ shaped curve, with increasing effects above or below a given temperature threshold, and is influenced by population characteristics (such as demographics, or underlying health condition), the built environment (for example housing type and quality), and any local adaptation (such as use of air conditioning, or behavioural change) (Gasparrini et al., 2015, Hajat et al., 2014). In the UK, pooled estimates derived from time-series epidemiological studies show that mortality increases by around 2.5% for every 1 °C above a daily mean threshold temperature of 18 °C, and for cold effects, there is around a 2.0% increase in mortality for every 1 °C below a daily mean threshold of 12 °C (Hajat et al., 2014, Vardoulakis et al., 2014). In this study, we use a high-resolution model simulation of the UHI in the West Midlands, combined with published exposure–response coefficients to 1) quantify the extent and intensity of the wintertime UHI in the West Midlands region, 2) estimate the impact of the wintertime UHI in reducing cold-related mortality, and 3) estimate how the health impacts may change in future, due to climate change.

## 2 Methods

We consider the winter period from mid-November 2009 to the end of March 2010, which was chosen as a particularly cold winter to compare with the hot summers examined in previous studies (Macintyre and Heaviside, 2019). We model temperatures using a meteorological model (Section 2.1) to quantify the UHI intensity and to give us exposure data in the form of daily mean 2 m air temperature, at 1 × 1 km horizontal resolution across the region. We then carry out a health impact assessment (HIA) based on the modelled exposure data and by applying published epidemiological relationships which relate daily mean temperature to increased risk of mortality, specific to this region and country (Section 2.2). This allows us to assess the impact of the UHI on temperature-related mortality.

### 2.1 Urban temperature modelling

Traditionally, the UHI intensity (difference between urban and rural temperatures) is quantified by taking the difference between observed temperature at urban and rural locations. However, weather station data has limited spatial coverage, and may have missing observation data. The use of meteorological computer simulations makes it possible to estimate the UHI intensity in space and time, by comparing temperatures simulated both with and without urban surfaces such as buildings and roads, and this is the approach used here. We simulate 2 m air temperature, and to quantify the UHI, we compare the temperatures between simulations that included urban land cover, with simulations whereby urban land categories were replaced by rural land cover, for the winter of 2009/10.

**Fig. 1:**
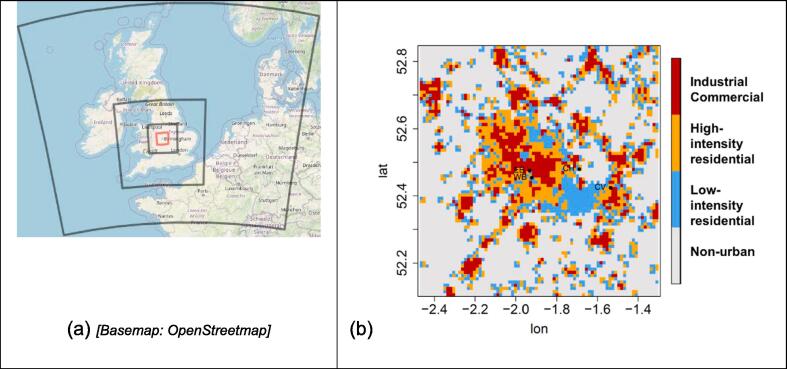
(a) Modelled domains in the WRF simulation. The central (red) box is the innermost domain, which is expanded in (b); (b) Urban categories used in the inner domain; the area covered is ~80 × 80 km. Lettered points refer to observation stations within the domain (refer to Table 2). (For interpretation of the references to colour in this figure legend, the reader is referred to the web version of this article.)

The WRF (Weather Research and Forecasting) model is a comprehensive prognostic weather forecasting and analysis model that has been used to investigate the impact of building interventions to modify the UHI at urban and regional scales; We used the WRF model version 3.6.1 (Chen et al., 2011) with four nested domains, adopting horizontal grid resolutions of 36 km, 12 km, 3 km, and 1 km, respectively, with two-way feedback between grids (Fig. 1). The model time-steps in each domain were 180, 60, 15 and 5 s, respectively, whilst meteorological variables, including 2 m air temperature were output at hourly intervals. Boundary conditions for the outermost domain were from the ERA-Interim reanalysis at 0.5° every 6 h (Dee et al., 2011), and there were 39 pressure levels above the surface, up to 1 hPa. We used a multilayer urban canopy scheme called Building Energy Parameterisation (BEP), which models the effect of buildings on horizontal and vertical energy and momentum fluxes inside and immediately above the urban street canyons, at a vertical resolution of 5 m, accounting for shading and radiation trapping in street canyons (Martilli et al., 2002). Information on building and road properties (e.g. building height, street canyon width, material properties such as albedo, thermal conductivity and heat capacity) is prescribed for three urban categories: industrial/commercial, high-intensity residential, and low-intensity residential, across the West Midlands region (Fig. 1b; Table 1) (Heaviside et al., 2015). The BEP scheme assumes a constant internal building temperature of 20 °C, which adds to the ground heat flux in the model through thermal conduction through the walls and roof; this does not explicitly consider other anthropogenic heat fluxes (such as from vehicles) during simulations. Land-surface data used as input to WRF for all domains were based on the US Geological Survey (USGS) 24-category land-use data, and for the inner domain we used two local datasets to generate the three separate urban categories (Owen et al., 2006). We used the Noah Land Surface Model (Noah-LSM), which is often coupled with an urban canopy scheme, and has four layers of soil moisture and soil temperature (Tewari et al., 2004). A tiled approach based on the urban fraction in each grid cell is used to divide energy fluxes between the BEP (urban) and Noah-LSM. The model has been previously run and validated for other periods using a similar configuration (Macintyre and Heaviside, 2019, Macintyre et al., 2018).

**Table 1:** (a) Details of default urban categories used in BEP (Building Energy Parameterisation); (b) General WRF model set-up details.

**(a)**			
**Category**	**1: Industrial/ commercial**	**2: High-intensity residential**	**3: Low-intensity residential**
Albedo (roof, wall, ground)	0.1989	0.1997	0.2027
Surface emissivity (roof, wall, ground)	0.9239	0.9274	0.9292
Average building height	25 m	15 m	10 m

**(b)**			

**Model setting**	**Option**		**Reference**

Long wave radiation	Rapid Radiative Transfer Model (RRTM)		Mlawer et al. (1997)
Short-wave radiation	Dudhia scheme		Dudhia (1989)
Boundary layer physics	Bougeault–Lacarrere (designed for use with BEP urban scheme)		Bougeault and Lacarrere (1989)
Urban physics	BEP urban scheme		Martilli et al. (2002)

The winter of 2009/10 saw prolonged periods of cold weather in the UK, dominated by easterly and north-easterly winds bringing cold air from northern Europe (Prior and Kendon, 2011). We ran the model from 14th November 2009 – 28th February 2010 and discarded the first day of simulation to remove the effect of initial conditions. We first ran the model with urban surfaces included, as described above (‘URBAN’) and evaluated the output against hourly observations from weather stations in the region (Fig. 1b, stations as lettered points), extracted from the Met Office Integrated Data Archive System (MIDAS) (Met Office 2012), as described below. We also ran a simulation replacing urban categories with surrounding rural cropland/pasture (‘RURAL’) for comparison. We quantified the UHI intensity in winter by taking the difference in temperature (population-weighted) between the URBAN and RURAL simulations.

We used hourly data from available MIDAS stations within the inner modelled domain for model evaluation, with modelled data extracted by bilinear interpolation from temperatures at the nearest four grid points in the 1 km grid spacing. The key statistics on the comparison between modelled temperatures for the ‘URBAN’ simulation and observed temperatures are shown in Table 2. Further figures for model comparison against observations can be found in the supplementary material (Fig. S1-3). The model compares well with observations, demonstrating that the model is able to capture diurnal variation in temperature at these sites, with correlation being 0.90 or higher (Table 2). The comparison is generally better for urban sites (Edgbaston, Coventry, Winterbourne); the comparison for a rural site (Coleshill, Table 2) is weaker than for urban sites, with 2 m air temperatures slightly underestimated. Quantifying the UHI based on the difference between observed temperatures at Edgbaston (semi-urban site) and Coleshill (rural site to the east of Birmingham) would give a mean UHI of 0.06 °C (0.21 °C at night, −0.09 °C daytime) for the whole winter period. However, this assumes that each site is representative of truly urban or rural conditions, and previous studies have shown that the Coleshill site may be influenced by the UHI, and the Edgbaston site is not representative of a fully urban area (Bassett et al., 2016, Heaviside et al., 2015).

**Table 2:** Model evaluation of 2 m temperature for the seasonal urban simulation based on MIDAS meteorological station observations.*

	Edgbaston (EB)		Coventry (CV)		Coleshill (CH)		Winterbourne (WB)	
	Observed	Modelled	Observed	Modelled	Observed	Modelled	Observed	Modelled
Mean (°C)	3.30	3.47	3.28	3.28	3.24	2.89	3.17	3.27
Standard deviation (°C)	3.63	3.63	3.80	3.90	3.94	4.08	3.74	3.70
RMSD‡ (°C)	–	1.41	–	1.62	–	1.85	–	1.69
Correlation coefficient	–	0.93	–	0.91	–	0.90	–	0.90

* Modelled data extracted by bilinear interpolation from temperatures at the nearest four grid points in the 1 km grid spacing. Numbers are calculated across all hourly values for the modelled period (15 Nov 09 – 28 Feb 10). In this context, standard deviation is indicative of the diurnal cycle across the modelled period.

‡ RMSD is the root mean square deviation, calculated from hourly values, as follows:$\mathrm{RMSD}=\sqrt{\frac{\sum_{n=1}^{N}{\left({\text{model}}_{n}-{\text{observed}}_{n}\right)}^{2}}{N}}$.

The coldest period within this winter ran from 16th December 2009 until the thaw on 15th January 2010; the week of 6th to 15th January saw overnight temperatures in England of −7 °C to −10 °C, and as low as −17.7 °C in Oxfordshire (Prior and Kendon, 2011). Model performance for this colder period is slightly weaker than for the whole winter period, in particular 6th – 15th January (supplementary Table S1, Fig. S4-6). At this time the observations first show very low temperatures (between −5 °C and −10 °C around 7th January) while the model is just below freezing (i.e. model is too warm). In contrast, from 10th January observed values hover around freezing, while modelled values are colder often by 2 to 3 °C (model too cold). There was significant snowfall on the night of 6th January, and then the 7th and 8th were bitterly cold, with a slight thaw on 10th, further snowfall on 12th and 13th, before thaw set in on 15th (Prior and Kendon, 2011). These few days from 10th to 15th January show weakest comparison with observations (Fig. S4). When meteorology is extremely locally-driven, such as during very cold stable periods, this can be associated with more stably stratified air, supressing mixing and giving a very small footprint to the station data, i.e. stations may represent much more local temperatures compared to what is resolved in the 1 km model. We find that weaker model-observation comparison of 2 m air temperature often coincides with lower wind speeds (Fig. S4). However, for 10th to 15th January the model shows reasonable comparison with windspeeds (Fig. S4) but the model is still too cold; this coincides with snowfall and snow on the ground, so it may be that local albedo effects from snow on urban surfaces or soil freezing are not adequately captured by the land and urban surface models. The model performance can also be dependent on different atmospheric conditions that prevail in different seasons and times of day, and this can be important when selecting model configuration (García-Díez et al., 2013), though a detailed analysis of this is complex and beyond the scope of this paper. The generally better comparison at urban sites highlights the importance of including urban surfaces when modelling temperature for health impact studies, as many climate change projections are unable to resolve urban areas due to the model resolution.

To estimate the effects of winter and summer UHIs in future climates, we used the UKCP18 seasonal temperature projections for the 2050s and 2080s, based on the central estimate (50th percentile) from the suite of probabilistic projections over land for RCP8.5 (25 km resolution), extracted for the West Midlands region (Table 3). These are applied as a temperature increment to the modelled values from the WRF model for all scenarios as a simple sensitivity study for future scenarios. Winter values are applied to the model simulations described in 2.1, and summer values are applied to simulated data for 1st June – 31st August 2006 using the same model set up, as described in Macintyre and Heaviside (2019).

**Table 3:** Seasonal mean temperature changes from the UKCP18 probabilistic projections for RCP8.5. Changes are relative to 1981–2010 baseline and based on 30-year time slices (2050s represents 2040–2069 and 2080s represents 2070–2099). Data is extracted for the West Midlands administrative region via the Met Office user interface web portal: https://ukclimateprojections-ui.metoffice.gov.uk.

	**2050 s**	**2080 s**
**Summer**	2.22 °C	4.66 °C
**Winter**	1.65 °C	3.01 °C

### 2.2 Health impact calculations

Our method to estimate the health impact of the winter UHI is to apply published exposure–response coefficients from an existing time-series epidemiological study that are specific to this population in this region of the UK. The exposure–response coefficients relate daily mean temperature exposure to increased risk of mortality. Increased risk of mortality corresponds to a value of ΔT (i.e. the difference between the daily mean temperature and the threshold temperature for effects).

We calculated the cold-related mortality over the winter period, under each scenario (URBAN, RURAL), by calculating daily cold-related mortality, $M_{i}$, and summing over all days, i, using the following method:

$$M_{i}=D_{i}\times {\mathrm{AF}}_{i}$$

$${\mathrm{AF}}_{i}=\left(\frac{{\mathrm{RR}}_{i}-1}{{\mathrm{RR}}_{i}}\right)$$

$${\mathrm{RR}}_{i}=e^{\left(b{\Delta T}_{i}\right)}$$

where $D_{i}$ is the all-cause mortality count for day i, AF (attributable fraction) is the fraction of daily mortality that can be attributed to the effects of cold exposure, defined by RR, which is the relative risk, depending on b, the slope of the exposure–response relationship (Vardoulakis et al., 2014), and ${\Delta T}_{i}$ is the mean daily population-weighted temperature for day i deducted from the threshold temperature for effects. We use the exposure–response coefficient derived in Vardoulakis et al. (2014), for the West Midlands region, which is approximately a 1.8% (95% CI: 1.6%, 2.1%) increase in mortality for every 1 °C decrease in daily mean temperature below the threshold of 11.7 °C (representing the 60th centile daily mean temperature for this region); the daily mean temperature is the mean temperature over that day and the preceding 27 days, representing the longer lag period for cold effects (as opposed to the 0–1 day lag for heat effects (Vardoulakis et al., 2014)), and so health effects are calculated from 12th December 2009 to 28th February 2010. Age-stratified exposure–response coefficients were available for the following age groups: 0–64, 65–74, 75–84, and 85+ years (Vardoulakis et al., 2014). Exposure is calculated based on gridded hourly 2 m air temperature output from the WRF model to calculate daily mean temperatures, and population weighting is by using a gridded 100 m residential population database (ONS, 2011, ONS, 2014.; 2015). This 100 m population database is combined with demographic information at Output Area^1^ (OA) level from the most recent census (ONS, 2011), following the method described in Macintyre et al. (2018). Daily all-cause mortality counts for 2009–10 were obtained from the Office for National Statistics. Differences between the URBAN and RURAL simulations were tested for significance at the 95% level using a *t*-test (two-sample, testing for equal variances) applied to daily mean population-weighted temperatures between the temperature simulations (URBAN, RURAL, described above in Section 2.1), and the estimated total daily cold-related mortality values.

We compared our results for cold-related effects during winter to heat-related impacts of the summer UHI based on simulations from a previous study (Macintyre and Heaviside, 2019), but to ensure the HIA is calculated over the same number of days in the summer and winter periods, we recalculated the summer HIA to ensure periods of equal length are compared. We therefore compare the winter UHI health effects from 12th December 2009 to 28th February 2010, and summer UHI health effects from 14th June – 31st August 2006. For heat effects of the summer UHI, we used the exposure–response coefficient also from Vardoulakis et al. (2014) for the West Midlands region, which is approximately a 2.5% (95% CI: 2.0%, 3.0%) increase in mortality for every 1 °C increase in daily mean temperature above a threshold of 17.7 °C (representing the 93rd centile daily mean temperature for this region). For estimation of future impacts, population size and demographics are held constant at present day levels to aid direct comparison of the seasonal effects, and daily mortality counts are not altered.

## 3 Results

### 3.1 The UHI in winter

**Fig. 2:**
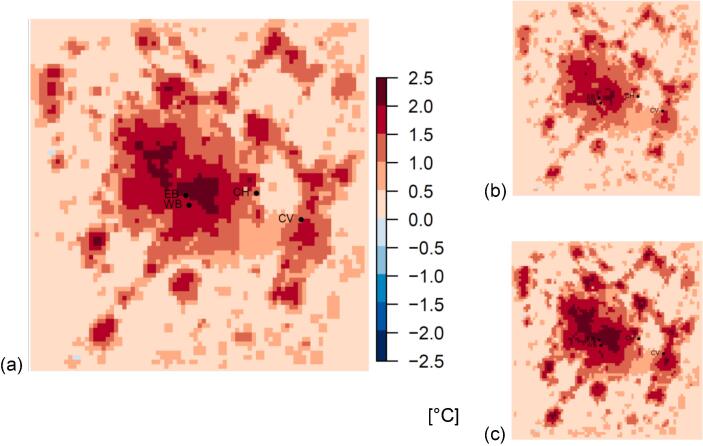
Urban Heat Island intensity for 15 Nov 09 – 28 Feb 10. (a) Mean 2 m temperature difference for the whole period; (b) daytime average (8 am – 8 pm); (c) night-time average (8 pm – 8 am). The UHI intensity in the center of the domain (Birmingham City center) in this figure is +2.4 °C (+2.3 °C daytime, +2.5 °C at night). Lettered points refer to observation stations (refer to Table 2).

The UHI intensity (calculated by taking the difference in 2 m temperature between the URBAN and RURAL simulations) over the winter period is shown in Fig. 2, with the average over all times shown (Fig. 2a) as well as broken down to day (Fig. 2b) and night time (Fig. 2c) averages.

**Fig. 3:**
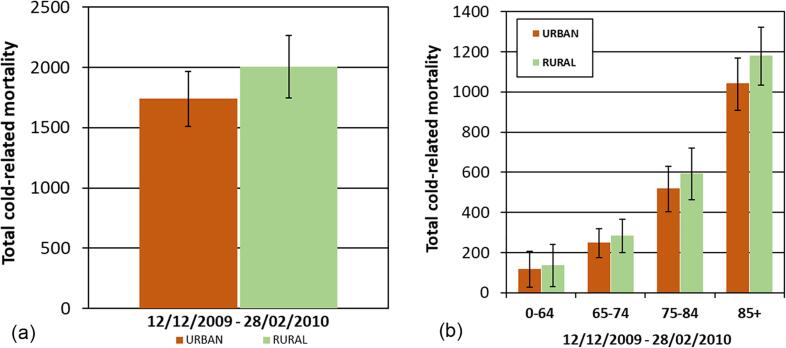
Cold-related mortality for the West Midlands for URBAN and RURAL simulations; (a) Total population and (b) age stratified. Bars represent 95% confidence intervals on the exposure–response relationship derived by Vardoulakis et al. (2014).

The mean population-weighted ambient temperature for the simulated winter period was 3.28 °C (3.50 °C during the day, and 3.01 °C at night, from the URBAN simulation), highlighting the low mean temperatures experienced during the winter 2009/10. Comparing the URBAN and RURAL simulations shows that the regional population-weighted mean UHI intensity is +1.5 °C (+1.54 °C at night, +1.36 °C during the day) (Fig. 2; Table 4); this difference between daily mean population-weighted 2 m air temperatures between the URBAN and RURAL simulations is statistically significant. The mean UHI intensity is highest in the city centre, being +2.4 °C on average over the winter period, and +2.5 °C at night (Fig. 2), and reached a maximum of +9.9 °C early one morning (15th January 2010 at 05:00). The characteristics of the UHI are similar between the day and night-times during this period (Fig. 2b,c); this contrasts with the typical summer UHI which tends to be more intense during the night than the day, as in summer the higher sun angles and longer days result in more heat absorbed by urban surfaces during the day, which is subsequently released at night. Additionally, the winter urban heat island is partially sustained by heat inside buildings that is conducted through the roof and wall surfaces; due to the low average temperatures over this period, there will be a continuous flux of heat from buildings driven by the indoor-outdoor temperature gradient.

**Table 4:** Temperature statistics for different model simulations for the ~11 week winter period (15 Nov 09 – 28 Feb 10). Values are population-weighted averages across the whole modelled domain, and broken down for day and night times. Numbers have been rounded from calculations with exact figures.

*Population weighted*	‘URBAN’ run T 2 m (°C)	‘RURAL’	
		T 2 m (°C)	ΔT (°C) (‘RURAL’ – ‘URBAN’)
Mean	3.28	1.80	−1.48
Day	3.50	2.15	−1.36
Night	3.01	1.46	−1.54

### 3.2 Health impact calculations – Deaths avoided in winter

Our results suggest that the UHI in winter increases temperatures in cities, which could reduce the number of cold-related deaths (Table 4; Fig. 3). For the RURAL simulation we estimate 2009 (95% CI: 1745–2265) cold-related deaths, and 1743 (95% CI: 1511–1969) for the URBAN simulation, suggesting that without the effect of the UHI in winter, the number of cold-related deaths would be higher by 266 (95% CI: 234–296), around 15%, over the period 12 December 2009 to 28 February 2010 (~11 weeks). This corresponds to a mean of ~25 cold-related deaths per day (RURAL) and ~22 per day (URBAN), suggesting that the UHI in winter has a potential small protective effect in this region. The greatest reduction in mortality is seen in the older age groups, as a larger proportion of baseline mortality is recorded in this group, coupled with the larger exposure–response coefficient (3.1% increase per degree for 85 + years, compared with 0.7% increase for 0–64 years) (Table 5).

**Table 5:** Estimated cold-related mortality for the winter period (12 December 2009 – 28 February 2010). Numbers in brackets represent the 95% confidence intervals based on the exposure–response coefficients.* Numbers are rounded from exact calculations.

	Age group	Estimated total number of cold-related deaths		
		URBAN simulation (95% CI)	RURAL simulation (95% CI)	Difference RURAL - URBAN (95% CI)
12 Dec 2009 – 28 Feb 2010	Total	**1743** (1511–1969)	**2009** (1745–2265)	**266** (234–296)
	0–64 yrs	**119** (27–206)	**139** (32–239)	**20** (5–33)
	65–74 yrs	**249** (174–319)	**284** (200–364)	**36** (26–45)
	75–84 yrs	**519** (403–630)	**594** (463–719)	**75** (60–89)
	85 + yrs	**1041** (909–1169)	**1182** (1034–1322)	**140** (125–153)

* Exposure-response relationship used from Vardoulakis et al. (2014) for the West Midlands region: RR 1.8% (CI: 1.6%–2.1%) increase in mortality for every 1 °C decrease in daily mean ambient temperature below 11.7 °C (below 60th centile). Age graded coefficients; 0.7% (0–64 years), 1.6% (65–74 years), 1.8% (75–84 years), 3.1% (85 + years).

To aid comparison with results for summer, we calculated heat related deaths for the summer of 2006 for the same number of days as the winter HIA (as described in Section 2.2 and Macintyre and Heaviside (2019)). We find the following heat-related deaths: for the URBAN simulation 267 (95% CI: 218–315); for the RURAL simulation 170 (95% CI: 139–202). Therefore, we estimate that the summer 2006 UHI contributed 96 (95% CI: 78–113) deaths, which can be expressed as 36% of heat-related mortality, over the period 14th June – 31st August 2006. In comparison, the winter 2009/10 UHI is associated with a 15% reduction of cold-related mortality (deaths avoided).

### 3.3 Impacts in the context of climate change

**Fig. 4:**
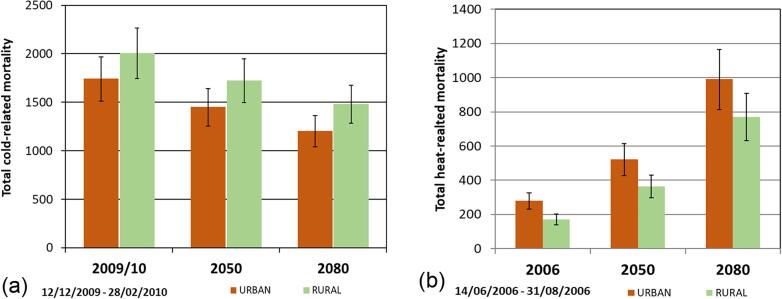
(a) Cold-related mortality and (b) heat-related mortality across the West Midlands for different climate scenarios and for URBAN and RURAL simulations, for an ~11 week period. 2050s and 2080s 50th percentile from the UKCP18 probabilistic projections (25 km) over land. Error bars show the range related to the 95% confidence intervals on the exposure–response coefficient (Vardoulakis et al., 2014).

The HIA for both URBAN and RURAL simulations was repeated with the same modelled data as described above, with addition of the temperature increment of projected changes from climate projections, as detailed in Table 3. As expected, estimated overall cold-related mortality decreases in the future (from 1743 to 1202 deaths by 2080s for URBAN; 541 fewer), and heat-related mortality increases (from 267 to 992 deaths by 2080s for URBAN; 725 more) (Fig. 4), tentatively suggesting a net increase in annual temperature-related mortality (for this population, time horizon and emissions scenario).

By comparing the HIA for URBAN and RURAL simulations, our results suggest that when including the impacts of climate change, the number of heat-related deaths associated with the UHI will increase from 96 in 2006, to 221 in 2080s, and due to the increase in overall heat-related mortality, and the use of a temperature threshold for health effects, the fraction associated with the UHI falls slightly from 36% in 2006 to 22% in 2080 s (Fig. 4b). For the winter simulation, cold-related mortality avoided due to the UHI increases slightly (266 deaths avoided in 2009, to 280 by 2080s, corresponding to 13% and 19% of overall cold-related mortality respectively), due to the greater relative reduction in mortality in the URBAN simulation from 2009/10 to 2080, compared with the changes for the RURAL simulation over the same time period.

Therefore, our results suggest that while currently the UHI has a net protective effect on heat- and cold-related mortality in absolute terms, in future the overall temperature-related mortality associated with the UHI will increase. This is because the change in the protective effect provided by the UHI in winter is smaller than the increase in heat-related deaths associated with the summer UHI.

## 4 Discussion and conclusions

Our analysis suggests that the mean winter population-weighted UHI intensity across the West Midlands is 1.5 °C in winter (reaching 2.3 °C in Birmingham city centre) and with a similar spatial pattern as in summer UHI (Fig. 2). This is comparable to other studies of the UHI for cities in northern mid-latitude climates in the US (1 °C to 4 °C) (Yang and Bou-Zeid, 2018), and for UK studies based on station measurements (0.6 °C to 1.7 °C) (Giridharan and Kolokotroni, 2009, Kershaw et al., 2010). To the best of our knowledge, this is the first attempt to model the winter UHI spatially and temporally by comparing simulations with and without urban surfaces, and to estimate the seasonal impacts on health for cold-related mortality. The UHI in winter exhibits less of a diurnal cycle than that in summer (shown by the similar UHI magnitudes in Fig. 2b,c); population weighted values for this region are 1.5 °C (1.54 °C at night; 1.36 °C daytime) in winter and 1.1 °C (1.8 °C at night; 0.5 °C daytime) in summer. In northern-hemisphere winter, the shorter days and lower sun angles lead to less solar insolation, and thus other sources of heat, such as anthropogenic and space-heating sources, are more dominant. The effect of heating from buildings is captured by the BEP scheme with buildings held at constant indoor temperature (representing space-heating), and this dynamically captures changes as the indoor-outdoor temperature gradient varies. For this particularly cold winter this will be more of a contributing factor to the UHI than solar insolation. However, an explicit anthropogenic heat flux is not included in the BEP model, which is a limitation of our study, although other studies using this type of modelling have shown that UHI intensification during particularly cold periods is independent of the background anthropogenic heat from sources other than buildings (such as vehicles) (Yang and Bou-Zeid, 2018). Anthropogenic sources of heat are challenging to quantify; Future work will include attempts to better approximate anthropogenic heat using assumptions about activity patterns, and vehicle types and usage, although detailed data is not always available (Hamilton et al., 2009).

The WRF model is a useful tool for simulating weather at the city/regional scale; using a multi-layer urban canopy scheme (BEP) to capture the effect of the built environment on energy and momentum fluxes in the lower atmosphere (accounting for shading and reflections by buildings) at sub-grid scale can provide suitable representation of how urban areas influence meteorology. The model comparison with observations is generally good (R^2^ > 0.9) but certain periods show disagreement, particularly the week leading up to 15th January 2010 (the end of a very cold period). This could be partly due to surface scheme model (the Noah-LSM) performance during periods where frozen ground or lying snow is present, which influence surface energy fluxes, and the occurrence of fog which can be challenging for models to predict and can lead to highly localised variation in energy fluxes (Bohnenstengel et al., 2014, Yang and Bou-Zeid, 2018). The model performance is generally better at urban stations with a small underestimate at the rural site here, but as we use population-weighting this should reduce the impact of this underestimate. Improving data on sources of anthropogenic heat for this area of the UK may also improve future studies of temperature effects in winter. Additionally, during particularly cold and atmospherically stable periods, conditions are likely to be highly locally driven, leading to station data representing a smaller area, potentially making it more challenging to compare with results interpolated from a gridded model.

The period we studied was particularly cold and snowy (Prior and Kendon, 2011). While climate projections show that winters will become generally milder, this does not preclude very cold periods from occurring; there is mixed evidence as to how blocking events (which often drive very cold weather) will change in future, and such events are challenging for atmospheric models to capture, particularly in winter seasons, which for northern mid-latitudes is when the majority of blocking events occur (Woollings et al., 2018). There is currently no clear consensus on whether cold waves would occur more often due to mid-latitude effects of Arctic warming (Blackport and Screen, 2020), and observations show decreasing cool spells in the northern midlatitudes have been getting milder over the last 50 years (van Oldenborgh et al., 2019). We have chosen a very cold winter here to investigate an extreme scenario in order to compare with the heatwave events previously studied. Future studies to investigate UHI behaviour in different years and regions should consider other winter scenarios, including milder ones representing a useful proxy for a potential future ‘typical’ winter.

Our HIA results suggest that the winter UHI reduced cold-related mortality by 15% between 12th December 2009 and 28th February 2010 (~11 weeks); we previously estimated that the summer UHI was associated with ~40% of heat related mortality for the same region. Cold mortality is higher than heat mortality in the UK each year, so although the proportion of cold deaths avoided due to the UHI is smaller than heat deaths attributed, in absolute terms, the winter UHI was associated with 266 fewer deaths, and the summer UHI with 96 more deaths. However, looking to a future climate projection, our results suggest that by the 2080s, the winter UHI will protect against 280 cold-related deaths, and the summer UHI will contribute to 221 heat-related deaths (Fig. 4), indicating increasing negative effects on annual mortality associated with the UHI in future. These results emphasise the differences in health impacts associated with the UHI between seasons in the UK and strengthens the argument for prioritising interventions to reduce summer urban heat in future, whilst not exacerbating cold impacts in winter. This is particularly important with rising temperatures in the UK. It is also worth noting that the protective effect of the UHI in milder winters may be different, so our results should be interpreted as a probable maximum effect. However, we acknowledge that population ageing, mortality counts, and adaptation are not accounted for, and this is intended as a sensitivity study for projected future changes in temperature.

Our HIA is based on time series regression modelling of ambient observed temperature and mortality, and we applied these results to temperatures simulated in a regional weather model run at high resolution; the different temperature data sources might introduce additional uncertainty, though to minimise this, simulated values are evaluated against station observations, and gridded temperature datasets (at the same resolution) are used for the timeseries study (Vardoulakis et al., 2014). The exposure–response coefficient used here is comparable to others in this region and other parts of the world, so it may be possible to generalise our HIA results to other areas, though differing thresholds and climates must be carefully considered in each case (Gasparrini et al., 2015). A fixed threshold is assumed for temperature-health effects, and we use region-specific exposure–response coefficients for heat and cold effects from the same epidemiological study (Vardoulakis et al., 2014); in this study the threshold for health effects from cold-exposure was assumed at the 60th centile of the annual temperature distribution (daily means), which is approximately the highest temperature in the coldest months of the year (December-March). As days with ambient temperatures below the threshold are common, this presents a challenge in defining cold thresholds, as increased risk of cold-related death can occur throughout much of the year, and different temperature-mortality coefficients and thresholds would yield different results (Arbuthnott et al., 2018). There is also inconsistency in what is defined as a cold spell (Ryti et al., 2016), and little evidence for cold-wave effects as with heatwaves (Barnett et al., 2012). Cold-exposure is associated with mortality over the course of a few weeks (0–27 day lag), which makes it challenging to identify health effects and infer causality, compared with heat effects which typically occur within one or two days (Ryti et al., 2016).

We have quantified the winter UHI and the associated impacts on cold-related mortality for a cold winter period, to compare to a hot summer period, including a simple sensitivity test for future climate. Our results suggest that even though there is presently a protective effect of the winter UHI (by reducing cold-related mortality in winter), due to the greater increase in heat-related mortality attributed to the summer UHI in a changing climate, overall impacts of the UHI on temperature-related mortality will become worse in future. Therefore, efforts to reduce summer urban heat are valuable, but should be carefully considered and assessed for any effects outside of the summer that might alter the protective effect of the winter UHI. Such assessments are currently very rare, but should be carried out to better inform climate adaptation strategies for health, to highlight any unintended consequences and avoid maladaptation.

## Declaration of Competing Interest

The authors declare that they have no known competing financial interests or personal relationships that could have appeared to influence the work reported in this paper.

## Footnotes

1 Output Areas (OA) are the lowest geographical level at which census estimates are provided. There are a total of 181,408 Output Areas in England and Wales, with an average population of 309 per OA.
