# Supplementary material for: The winter urban heat island: Impacts on cold-related mortality in a highly urbanized European region for present and future climate
Source: Environ Int. 2021 Sep;154:106530. doi: 10.1016/j.envint.2021.106530 (PMC8543073; doi:10.1016/j.envint.2021.106530)
Supplement: Supplementary data 1 [file mmc1.pdf]

Supplementary Information

Supplementary information for “The winter urban heat island: impacts on cold-related mortality in a highly urbanized European region for present and future climate”

1. Model evaluation

Coleshill

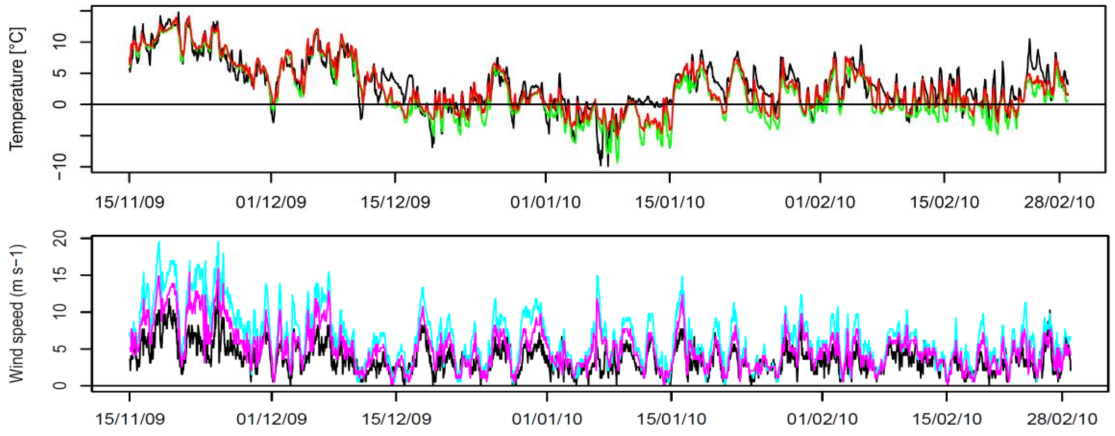

Coventry

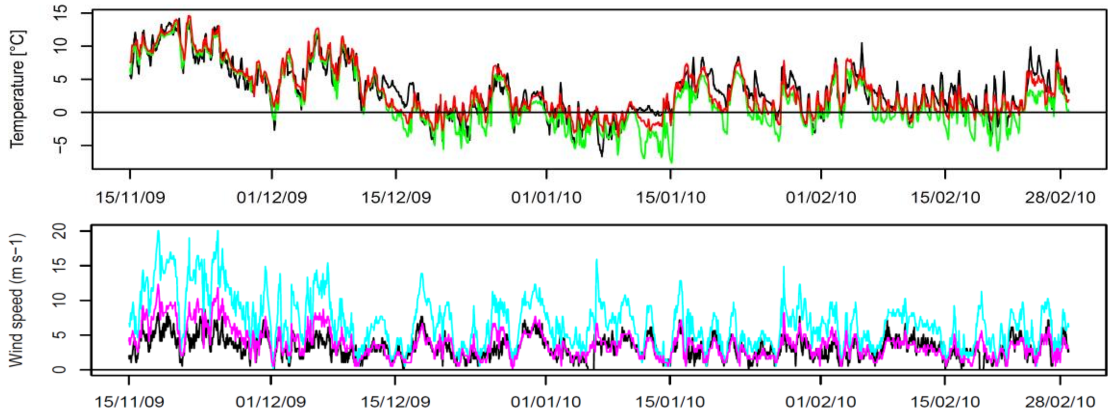

Edgbaston

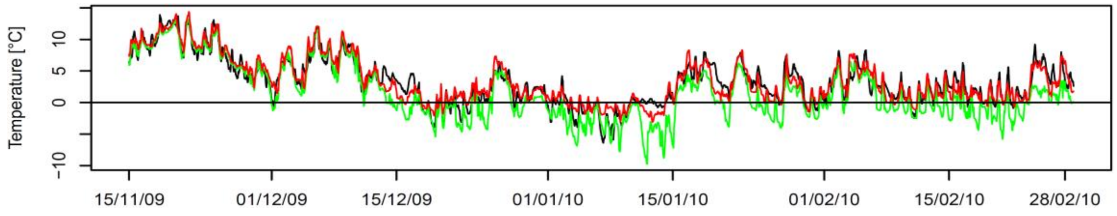

[wind speed observations not available]

Winterbourne

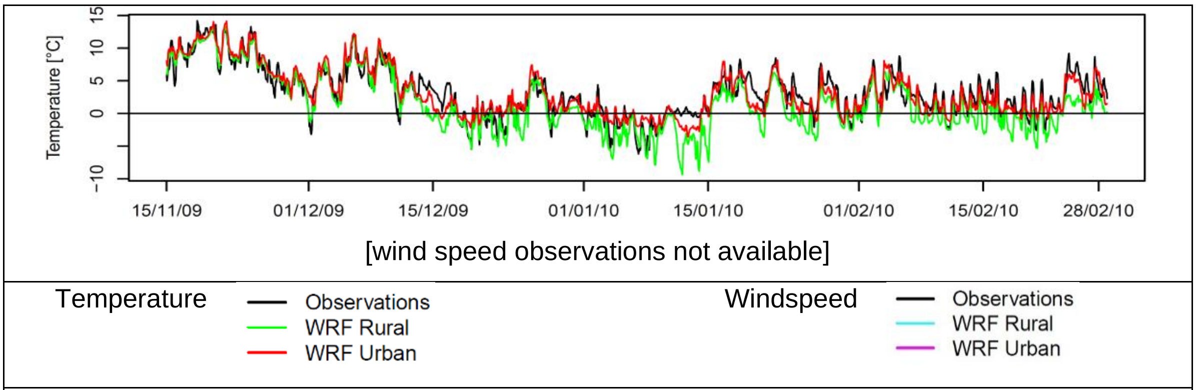

Fig. S1. Timeseries comparison for observations (black) and WRF urban simulation (red) for hourly 2 m temperature.

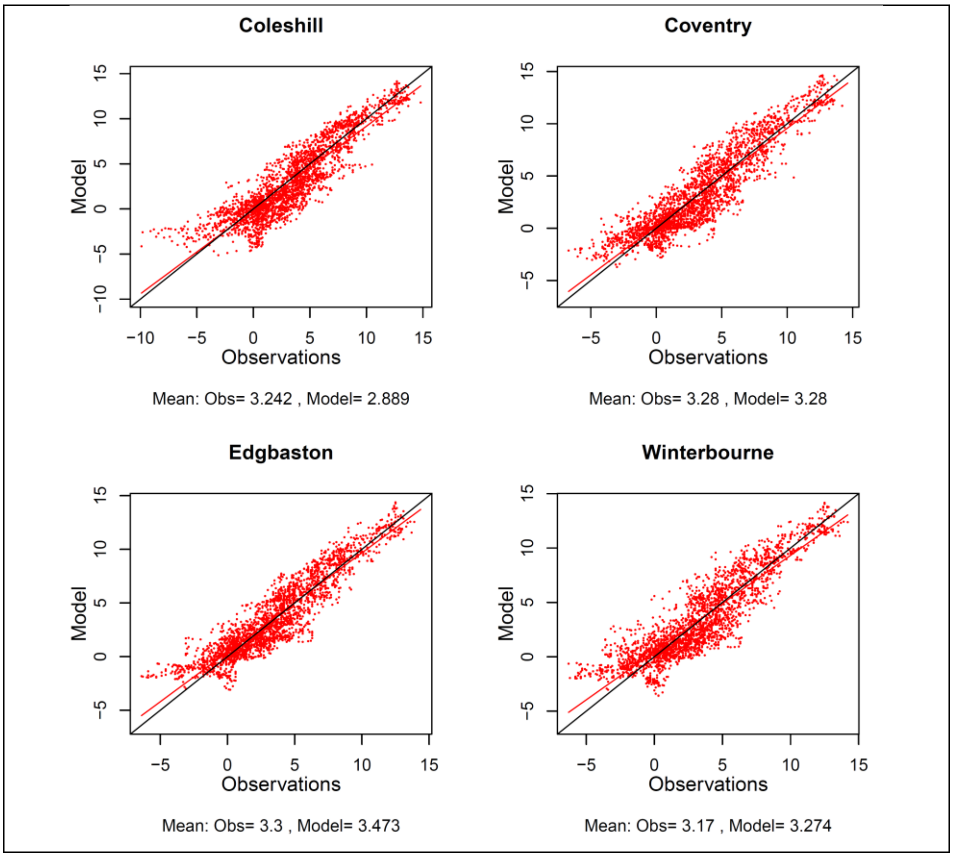

Fig. S2. Scatter plots of modelled vs observed hourly 2 m air temperature.

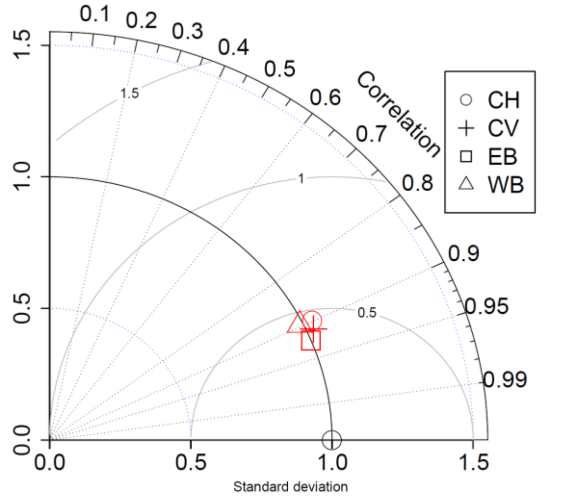

Fig. S3. Taylor plot for observed vs modelled values for the ‘urban’ WRF simulation.

2. Coldest period from 16 December 2009 – 15 January 2010.

Comparison for the coldest period is weaker than for the whole winter period. The model particularly struggles for the 6th – 15th January. At this time the observations first show very low temperatures (between −5°C and −10°C around 7th January), and then show values hovering around freezing, while modelled values are colder often by 2-3°C. There was snowfall on the night of 6th January, and then the 7th and 8th were bitterly cold, with a slight thaw on 10th and then further snowfall on 12th and 13th, before thaw set in on 15th (Prior and Kendon 2011). The comparison is better for urban sites (Table S1, sites EB and CH).

Including urban surfaces (Fig.S4, S5, red line for temperature, purple line for windspeed) improves the model compared to rural land cover only (green line).

The period from 10th -15th January shows poorest comparison to observations, and it is therefore difficult to know if the UHI as quantified here by taking the difference between the URBAN and RURAL simulations is reliable. The comparison for windspeeds for this period appears reasonable; the presence of snow at this time and the associated effects of lying snow and the albedo effect of this on surface energy fluxes might be a reason for the mismatch in temperatures. The results show that including urban land cover greatly improves the model performance for both 2 m air temperature and windspeeds, reinforcing the importance of adequate representation of surface properties when modelling near-surface conditions such as 2 m air temperature.

Table S1. Model evaluation of 2 m temperature for the cold period 16 Dec 2009 to 15 Jan 2010, based on MIDAS meteorological station observations*.

	Edgbaston (EB)		Coventry (CV)		Coleshill (CH)		Winterbourne (WB)	
	Observed	Modelled	Observed	Modelled	Observed	Modelled	Observed	Modelled
Mean (°C)	0.18	0.71	0.15	0.41	0.04	-0.25	0.02	0.48
Standard deviation (°C)	2.27	1.93	2.37	2.09	2.75	2.31	2.36	1.95
RMSD‡ (°C)	–	1.52	–	1.64	–	2.00	–	1.79
Correlation coefficient	–	0.78	–	0.74	–	0.70	–	0.69

*All are calculated across all hourly values for the modelled period (16/12/2009 – 15/01/2010). In this context, standard deviation is indicative of the diurnal cycle across the modelled period.

‡RMSD is the root mean square deviation, calculated from hourly values, as follows: $RMSD = \sqrt{\frac{\sum_{n=1}^{N}(\text{model}_n - \text{observed}_n)^2}{N}}$

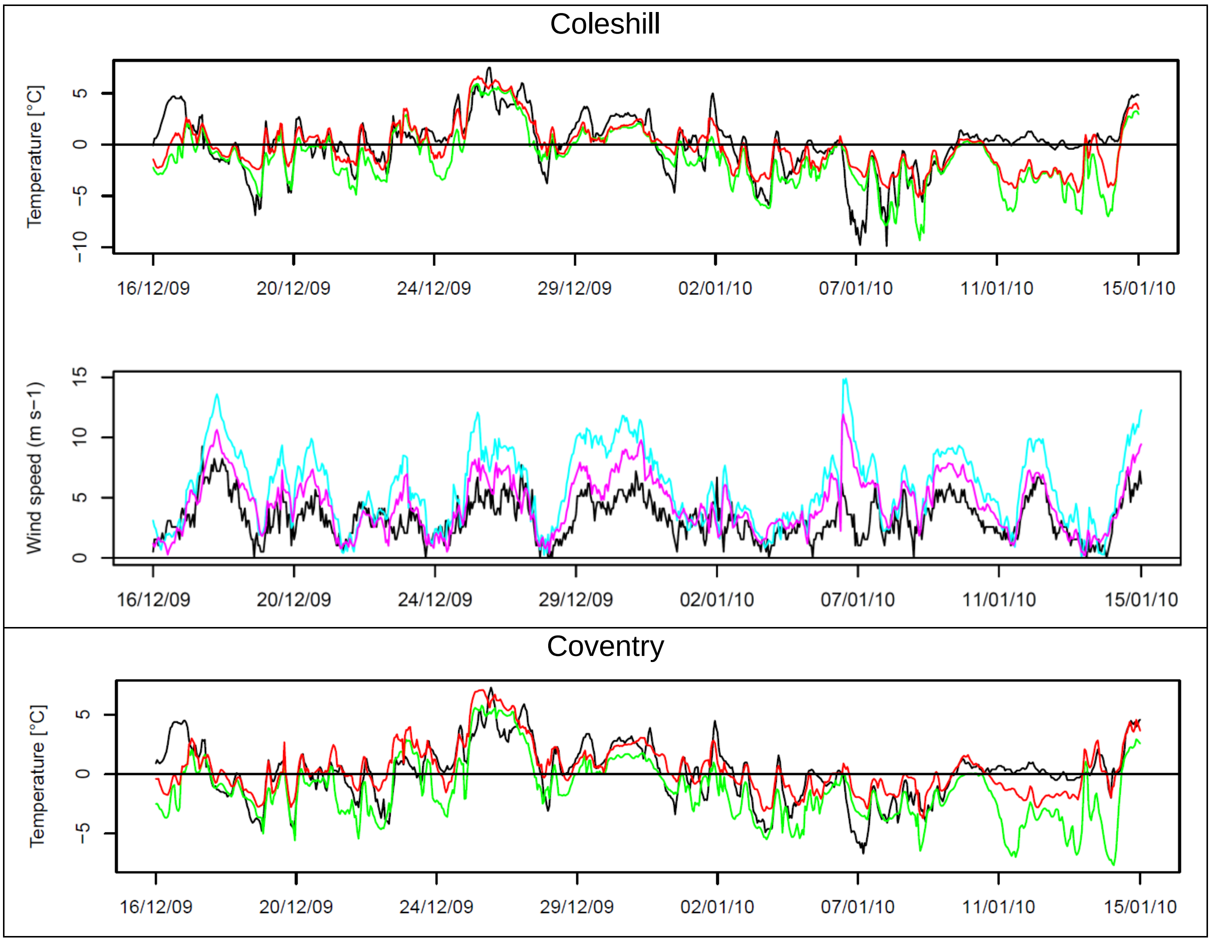

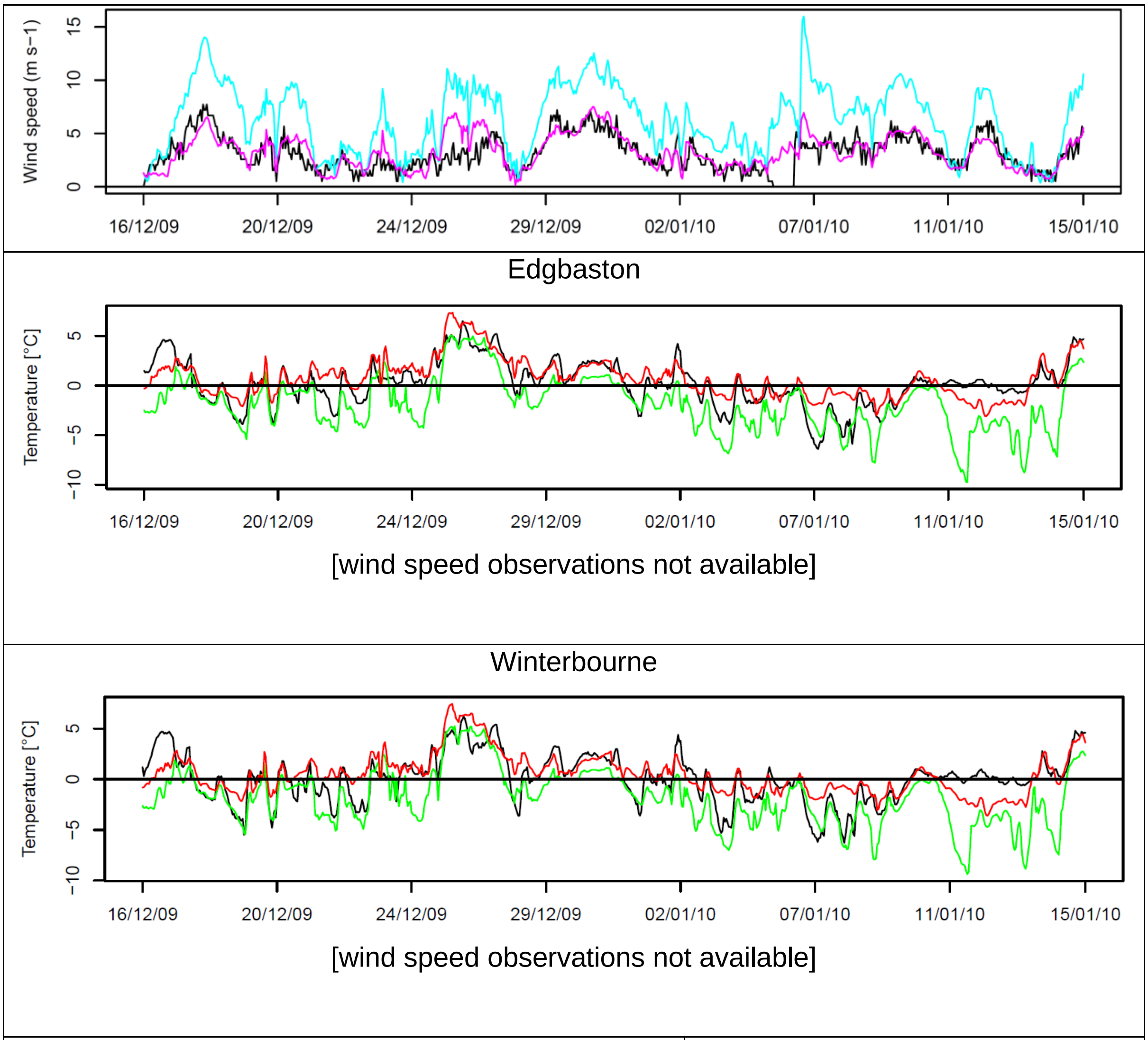

Figure S4: Model-observation comparison of 2m air temperature and windspeed (where data is available) for the coldest period 16/12/2009 – 15/01/2010.

temperature	windspeed
Observations	Observations
WRF Rural	WRF Rural
WRF Urban	WRF Urban

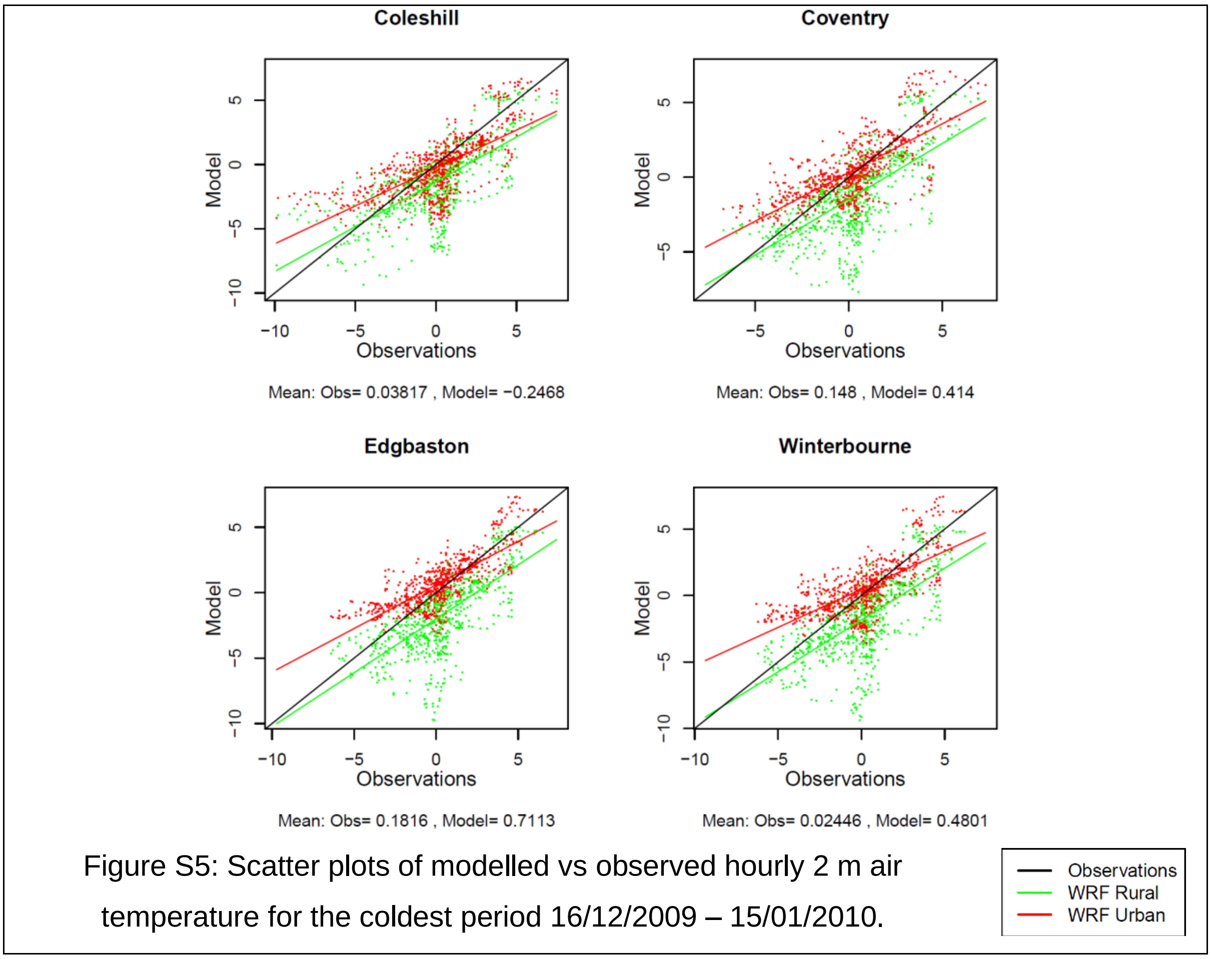

Figure S5: Scatter plots of modelled vs observed hourly 2 m air temperature for the coldest period 16/12/2009 – 15/01/2010.

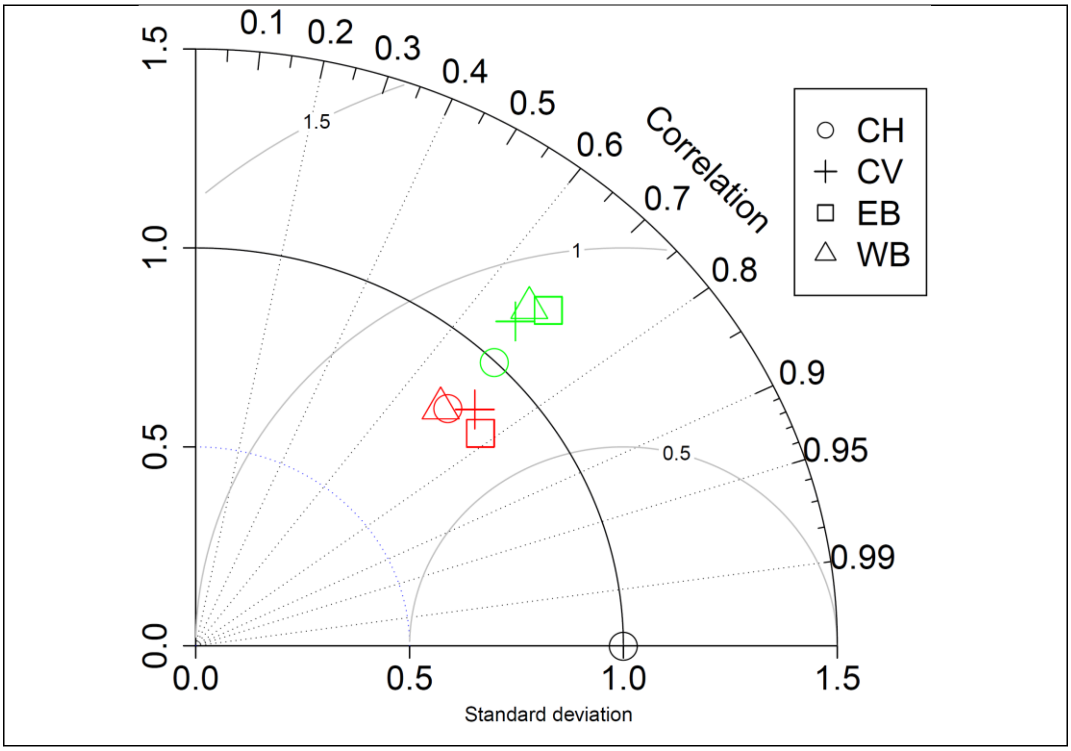

Figure S6: Taylor diagram of 2 m air temperature for the coldest period (16/12/2009 – 15/01/2010).

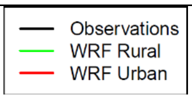

References

Prior, J.; Kendon, M. The UK winter of 2009/2010 compared with severe winters of the last 100 years. 2011;66:4-10
